# Identifying Novel Copy Number Variants in Azoospermia Factor Regions and Evaluating Their Effects on Spermatogenic Impairment

**DOI:** 10.3389/fgene.2019.00427

**Published:** 2019-05-07

**Authors:** Ran Zhou, Jian Cheng, Dingyuan Ma, Jianxin Tan, Yuguo Wang, Ping Hu, Zhengfeng Xu

**Affiliations:** State Key Laboratory of Reproductive Medicine, Department of Prenatal Diagnosis, Nanjing Maternity and Child Health Care Hospital, Women's Hospital of Nanjing Medical University, Nanjing, China

**Keywords:** male infertility, Y chromosome, azoospermia factor regions, spermatogenic failure, copy number variants

## Abstract

Microdeletions in Y-chromosomal azoospermia factor (AZF) regions have been regarded as the risk factor of spermatogenic failure (SF). However, AZF-linked duplications or complex copy number variants (CNVs) (deletion + duplication) were rarely studied. In this study, we performed multiplex ligation-dependent probe amplification (MLPA) analysis on 402 fertile healthy male controls and 423 idiopathic infertile SF patients (197 azoospermia and 226 oligozoospermia) in Han Chinese population. In total, twenty-four types of AZF-linked CNVs were identified in our study, including eleven novel CNVs (one deletion, seven duplications, and three complex CNVs). Our study revealed that AZFc-linked duplications and the instability of Y chromosome might be associated with spermatogenesis. Besides, the complex CNVs (b2/b3 deletion + *DAZ*1/2 duplication) were confirmed to increase genetic risks for SF in Han Chinese population. This study illustrated a spectrum of AZF-linked CNVs and presented valuable information for understanding the clinical significance of AZF-linked CNVs in male infertility.

## Introduction

Infertility affects an estimated 15% of couples globally at childbearing age, and male infertility is directly or indirectly responsible for about 50% of cases involving reproductive-age couples with fertility-related issues (Agarwal et al., 2015). The majority of infertile males were diagnosed with spermatogenic failure (SF) (Lo Giacco et al., 2014).

Y-chromosome microdeletions (YCM) are the second most common genetic etiology for SF after the Klinefelter syndrome (Krausz et al., 2014). In YCM, azoospermia factor (AZF) [AZFa, AZFb, AZFc(b2/b4)] deletions have resulted in SF and testing these deletions had significant clinical values both in diagnosis and prognostication of testicular sperm retrieval in azoospermic men (Pryor et al., 1997; Rozen et al., 2012), while the clinical significance of partial AZFc deletions (gr/gr, b2/b3, and b1/b3) varies in different studies (Lu et al., 2009; Bansal et al., 2016; Krausz and Casamonti, 2017). However, all previous studies on AZF-linked copy number variants (CNVs) have mainly focused on deletions rather than duplications or complex CNVs (deletions + duplications).

Owing to frequent non-allelic homologous recombination (NAHR) of AZF region, the majority of CNVs are found on Y chromosome among all human chromosomes (Freeman et al., 2006). Identifying AZF-linked CNVs have been showed to be valuable for finding the genetic etiology of SF, as well as evaluating the rate of sperm recovery after testicular sperm extraction (TESE) (Krausz et al., 2014). Traditional methods to identify AZF-linked CNVs were based on polymerase chain reaction (PCR) of sequence-tagged site (STS) markers (STS-PCR), however, STS-PCR could only determine AZF-linked deletions. To date, some AZF-linked duplications or complex CNVs have been reported (Giachini et al., 2008; Lu et al., 2011; Saito et al., 2015). However, the precise frequency and clinical significance of these CNVs have remained unclear in patients with SF.

With the development of molecular techniques, such as multiplex ligation-dependent probe amplification (MLPA) and array comparative genomic hybridization (aCGH), researchers were able to simultaneously identify multiple CNVs. Compared with aCGH, the MLPA method was relatively simple and inexpensive, and it has been suggested as a valuable technique for diagnosing genetic diseases (Quarello et al., 2012; Massalska et al., 2013; Mutlu et al., 2018).

To identify novel AZF-linked CNVs (especially duplications and complex CNVs) and their significance in spermatogenesis, we performed MLPA analysis on 402 fertile healthy male controls and 423 idiopathic infertile SF patients (197 azoospermia and 226 oligozoospermia) in Han Chinese population.

## Materials and Methods

### Study Subjects

Subjects were recruited from the Nanjing Maternity and Child Health Care Hospital (Nanjing, Jiangsu province, China) between July 2016 and September 2017. This study was approved by the Ethics Committee of Nanjing Maternity and Child Health Care Hospital. All procedures involved in this study were conducted in accordance with the Declaration of Helsinki.

Semen analyses were performed based on World Health Organization criteria (2010) with the reference values parameters for semen volume of ≥ 1.5 mL, pH ≥ 7.2, total sperm count of 39 × 10^6^ per ejaculate, sperm concentration of 15 × 10^6^/mL, sperm total motility of 40%, and sperm with a normal morphology of 4% (Cooper et al., 2010), and each subject was examined twice to ensure the reliability of the results. In addition to semen analyses, some comprehensive andrological examinations including a series of physical examinations, scrotal ultrasound, hormone analysis and karyotype analysis were also performed. Those with varicocele, cryptorchidism, orchitis or abnormal karyotype (such as 47, XXY) were excluded from this study.

The male infertile cases, who sought treatment in the infertility clinic, were recruited into this study by a retrospective design. These cases have no child and diagnosised with idiopathic non-obstructive azoospermia (NOA) (no sperm in the ejaculate) or oligozoospermia (total sperm count < 39 × 10^6^ per ejaculate). To distinguish between NOA and OA (obstructive azoospermia), only those azoospermic cases with soft and small testis (total testicular volume <30 ml), elevated follicular stimulating hormone in plasma were included. The controls, recruited from the same hospital during the same period, were fertile males who had normal total sperm count per ejaculate, sperm concentration, motility, morphology and had fathered at least one child without assisted reproductive. Twenty-nine infertile subjects (out of 452) were excluded, including 4 with OA, 5 with cryptorchidism, 8 with varicocele, 2 with orchitis and 10 with abnormal karyotype (7 of them with 47, XXY). Finally, 402 fertile male controls and 423 infertile patients were recruited in this study.

All the participants were genetically unrelated ethnic Han-Chinese. Some influencing factors [e.g., age, body mass index (BMI), and smoking] were ruled out between patients and controls. All participants were informed about the purpose of the study, and signed the written informed consent for publication of this original research as well.

### MLPA Analysis

MLPA analysis was performed using SALSA MLPA probe-mix kit P360-B1 (MRC-Holland, Amsterdam, the Netherlands) containing 42 specific probes for AZF regions (Supplementary Figure S1). Briefly, 7 ul of sample DNA (20 ng/ul) were denatured for 5 min at 98°C and subsequently cooled to 25°C. Next, 5 ul denatured DNA were mixed with 3 ul hybridization master mix and heated for 1 min at 95°C, then 18 h at 60°C. After hybridization reaction, 32 ul ligase-65 master mix were added and incubated for 15 min at 54°C and 5 min for 98°C, then pause at 20°C. The ligation products were then mixed with polymerase master mix for PCR reaction. The PCR products were analyzed by ABI 3,500 using 50 cm capillaries and POP-7 polymers (Applied Biosystems, Foster City, CA, USA) and the injection mixture containing 0.9 ul PCR products, 0.1 ul LIZ 500 size standard and 9 μl HiDi formamide. The ABI 3,500 run conditions were as follows: injection voltage = 1.6 kVolt, injection time = 8 s, oven temperature = 60°C, run voltage = 19.5 kVolts, and run time = 1330 s. The relative peak area of each probe was calculated by dividing the actual peak area of the subject by the average of that of five reference samples using Coffalyser.Net Software (MRC-Holland, Amsterdam, the Netherlands). The 30% increase or decrease of the relative peak area of the probe showed duplication or deletion of the targeted region, respectively.

### PCR of Sequence-Tagged Site (STS) Markers (STS-PCR)

Results of MLPA analysis were compared with those of conventional STS-PCR based on ten STS markers in AZF region (sY84 and sY86 for AZFa, sY127 and sY134 for AZFb, sY254, sY255, sY1192, sY1191, sY1291 and sY1189 for AZFc) recommended by EAA/EMQN practice guidelines (Krausz et al., 2014).

### Real-Time Quantitative PCR (qPCR)

To validate those AZF-linked CNVs not confirmed by STS-PCR, we performed real-time quantitative PCR (qPCR). Primer sequences designed for target regions were listed in Supplementary Table S2. The *ACTB* gene was employed as a reference. The qPCR was performed in a total volume of 20 μL consisting of 10 μL AceQ Universal SYBR qPCR Master Mix (Vazyme, China), 0.4 μL of 10 μM each primer (forward and reverse), 2 μL genomic DNA (10 ng/μL) and 7.2 μL nuclease-free water. The PCR reaction were run in ABI StepOnePlus^TM^ (Life Technologies, USA) based on the following program: 95°C for 5 min for initial denaturation, followed by 40 cycles (95°C for 10 s and 60°C for 30 s) for amplification and fluorescence detection, and a cycle (95°C for 15 s, 60°C for 60 s, and 95°C for 15 s) for melt curve analysis. Data analysis was performed using StepOneTM Software based on the ^ΔΔ^CT method. All samples were detected in triplicates for qPCR analysis.

### Statistical Analysis

In this study, χ^2^ and Fisher's exact tests were applied to compare the statistical differences in the frequencies of CNVs between the patients and controls using SPSS 20.0 software (IBM, Armonk, NY, USA). *P*-value < 0.05 was statistically considered significant. We did not correct for multiple testing considering that the sample size for those novel AZF-linked CNVs were relatively small and adjustments for making multiple comparisons might miss possibly important findings.

## Results

### Characteristics of the Study Population

In this study, we recruited 402 fertile healthy male controls and 423 infertile SF patients (197 azoospermia and 226 oligozoospermia). According to the achieved results, we found that there was no significant difference between the control group and the SF group in selected characteristics including age, BMI, and smoking (Table 1).

**Table 1:** Study population information of controls and patients.

**Variables**	**Frequency**	**Controls**	**Patients**					
		**Normozoospermia(*n* = 402)**	**Spermatogenic failure^a^(*n* = 423)**	***P***	**Azoospermia(*n* = 197)**	***P***	**Oligozoospermia(*n* = 226)**	***P***
Age (years)^b^	–	28.52 ± 3.72	28.45 ± 3.55	9.52 × 10^−1^	29.4 ± 3.54	4.81 × 10^−1^	27.67 ± 3.45	4.61 × 10^−1^
BMI^b^	–	22.98 ± 3.00	21.73 ± 2.82	1.35 × 10^−1^	21.86 ± 3.30	3.04 × 10^−1^	21.63 ± 2.46	1.40 × 10^−1^
Smoking^c^	Ever	23 (57.96%)	251 (59.34%)	6.90 × 10^−1^	114 (57.87%)	9.82 × 10^−1^	137 (60.62%)	5.26 × 10^−1^
	Never	169 (42.04%)	172 (40.66%)		83 (42.13%)		89 (39.38%)	

a The sum of azoospermia and oligozoospermia;

b The difference in age and BMI between controls and patients were tested using independent-sample T-test, data were expressed with mean ± SD (standard deviation);

c *The frequency difference in smoking between controls and patients were tested using χ^2^ test; P-values < 0.05 were considered significant; BMI, body mass index*.

### Distribution of Known and Novel Deletions in AZF Regions and Their Effects on SF

**Figure 1:**
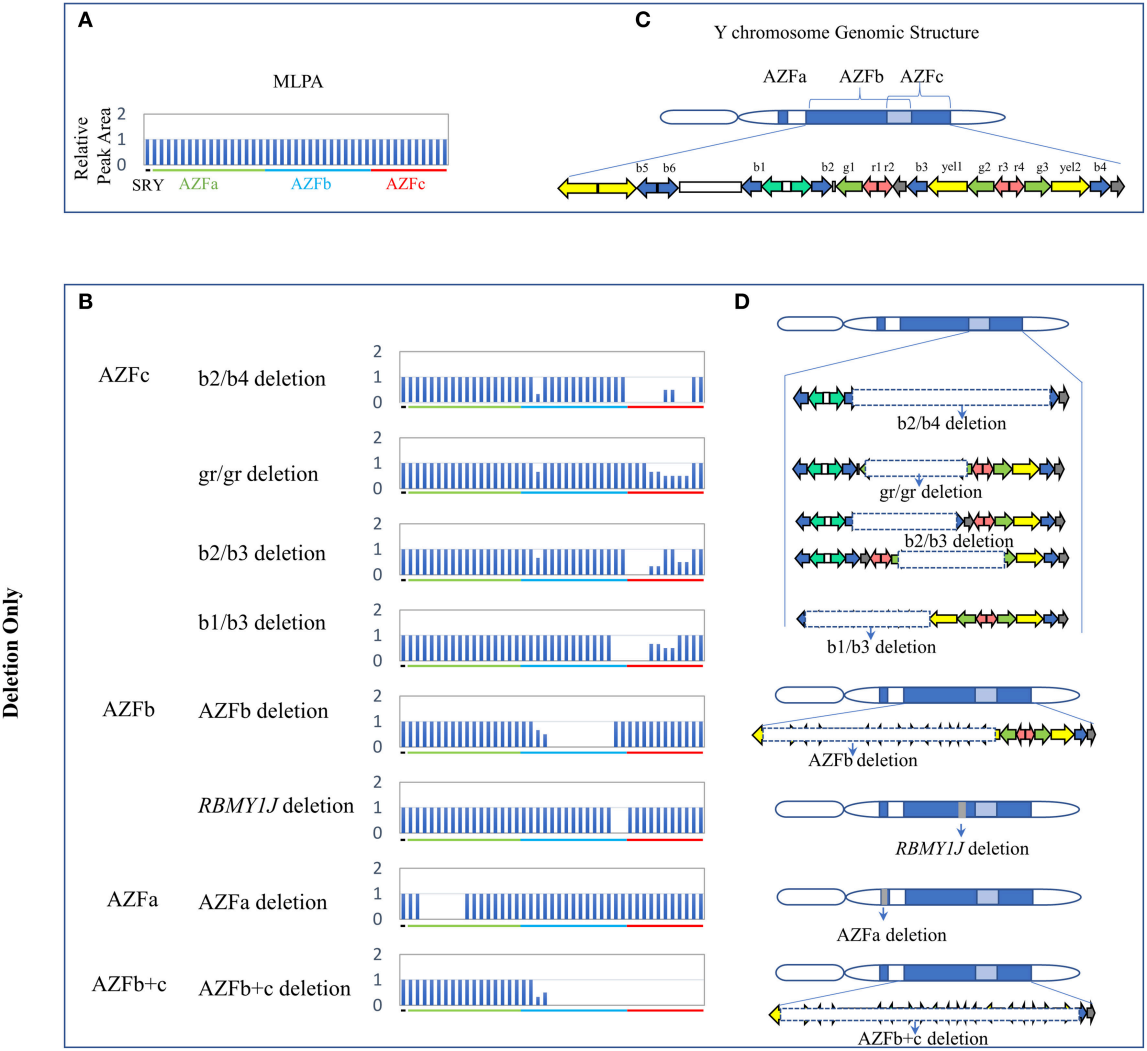
CNVs (Deletion Only) in AZF regions of the human Y chromosome identified by MLPA. **(A,B)** Results of MLPA analysis. The relative peak area of each probe was calculated by dividing the actual peak area of the subject by the average of that of five reference samples. Decreased relative peak areas mean copy-number deletion. **(C,D)** Corresponding structures and rearrangements of AZF regions.

Overall, twenty-four types of AZF-linked CNVs (eight deletions, eleven duplications and five complex CNVs) were identified in our study population. Among eight deletions, four classical deletions (AZFa deletion, AZFb deletion, AZFc complete deletion (b2/b4 deletion), and AZFb + c deletion) recommended by EAA/EMQN practice guidelines (Krausz et al., 2014) were identified by MLPA analysis and verified by STS-PCR (Figures 1, 4). These four types of deletions were identified in SF patients, but not observed in controls (Table 2). Consistent with the previous reports, the classical deletions were the genetic causes of SF (Krausz et al., 2014).

**Table 2:** Distribution of known and novel deletions in AZF regions and their effects on SF.

**Deletion types**			**Controls**			**Patients**								
			**Normozoospermia (*****n*** **=** **402)**			**Spermatogenic failure (*****n*** **=** **423)**			**Azoospermia (*****n*** **=** **197)**			**Oligozoospermia (*****n*** **=** **226)**		
			***n***	**OR**	***P***	***n***	**OR (95%CI)**	***P***	***n***	**OR (95%CI)**	***P***	***n***	**OR (95%CI)**	***P***
Deletion only	AZFc	b2/b4 del	0	1.00	–	23	–	–	9	–	–	14	–	–
		gr/gr del	20	1.00	–	32	1.56	1.29 × 10^−1^	14	1.46	2.92 × 10^−1^	18	1.65	1.35 × 10^−1^
							(0.88–2.78)			(0.72–2.96)			(0.86–3.19)	
		b2/b3 del	24	1.00	–	16	0.62	1.47 × 10^−1^	8	0.67	3.32 × 10^−1^	8	0.58	1.89 × 10^−1^
							(0.32–1.18)			(0.29–1.51)			(0.26–1.31)	
		b1/b3 del	2	1.00	–	5	2.39	2.99 × 10^−1^	0	–	–	5	4.53	7.30 × 10^−2^
							(0.46–12.40)						(0.87–23.52)	
	AZFb	AZFb del	0	1.00	–	1	–	–	1	–	–	0	–	–
		*RBMY1J* del^*^	0	1.00	–	1	–	–	0	–	–	1	–	–
	AZFa del		0	1.00	–	2	–	–	2	–	–	0	–	–
	AZFb + c del		0	1.00	–	7	–	–	7	–	–	0	–	–
	Classical del (b2/b4 del/AZFb del/AZFa del/AZFb + c del)		0	1.00	–	33	–	–	19	–	–	14	–	–

* *indicated that the CNV was not reported previously; del, deletion; P-values < 0.05 were considered significant*.

The frequency of three AZFc partial deletions (gr/gr, b2/b3, and b1/b3) identified by MLPA and validated by STS-PCR showed no significantly statistical difference between the patients and the controls (Table 2), suggesting that these partial deletions might not considered to be genetic risk factors for SF in our study population.

Besides, we found a novel and *de novo* AZFb partial deletion (*RBMY1J* deletion) in a patient with oligozoospermia whose father has a normal *RBMY1J* copy in the same region (Table 2). The *de novo* deletion might be associated with oligozoospermia, and further study is required to confirm whether the *RBMY1J* deletion may cause the disease.

### Distribution of Known and Novel Duplications in AZF Regions and Their Effects on SF

**Figure 2:**
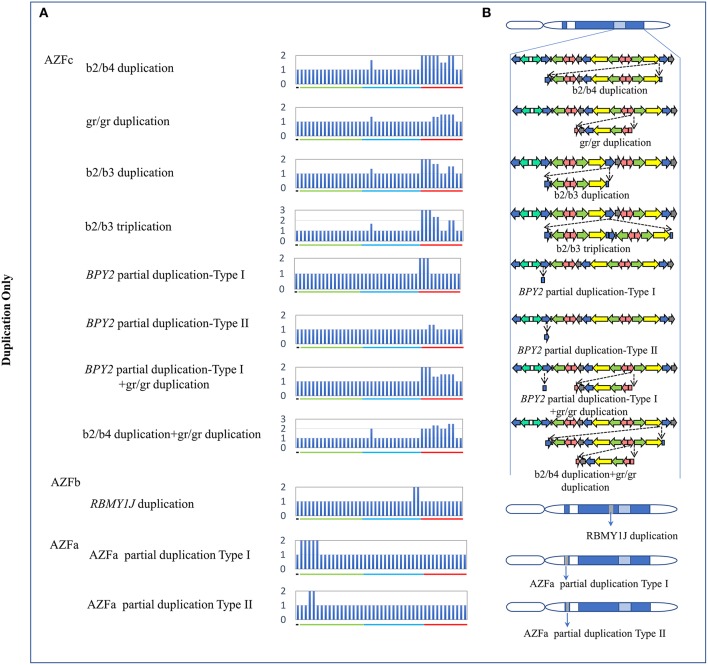
CNVs (Duplication Only) in AZF regions of the human Y chromosome identified by MLPA. **(A)** Results of MLPA analysis. The relative peak area of each probe was calculated by dividing the actual peak area of the subject by the average of that of five reference samples. Increased relative peak areas mean copy-number duplications. **(B)** Corresponding structures and rearrangements of AZF regions.

The traditional STS-PCR method could only detect AZF-linked deletions, while MLPA method could detect not only deletions, but also duplications. In this study, we identified eleven types of AZF-linked duplications in 3.98% of controls (16/402) and 7.09% of SF patients (30/423). Most of duplications (72.7%, 8/11) were located in the AZFc region likely owing to the frequent non-allele homologous recombination (NAHR) in this region (Skaletsky et al., 2003). Seven novel duplications, four in AZFc region, one in AZFb region, and two in AZFa region, were identified in our study (Figure 2, Table 3).

**Table 3:** Distribution of known and novel duplications in AZF regions and their effects on SF.

**Duplications types**			**Controls**			**Patients**								
			**Normozoospermia (*****n*** **=** **402)**			**Spermatogenic failure (*****n*** **=** **423)**			**Azoospermia (*****n*** **=** **197)**			**Oligozoospermia (*****n*** **=** **226)**		
			***n***	**OR**	***P***	***n***	**OR (95%CI)**	***P***	***n***	**OR (95%CI)**	***P***	***n***	**OR (95%CI)**	***P***
Duplication only	AZFc	b2/b4 dup	2	1.00	–	3	1.43	6.97 × 10^−1^	0	–	–	3	2.69	2.80 × 10^−1^
							(0.24–8.60)						(0.45–16.22)	
		gr/gr dup	5	1.00	–	13	2.52	8.20 × 10^−2^	7	2.93	6.99 × 10^−2^	6	2.17	2.06 × 10^−1^
							(0.89–7.13)			(0.92–9.34)			(0.65–7.18)	
		b2/b3 dup	1	1.00	–	4	3.83	2.31 × 10^−1^	3	6.20	1.15 × 10^−1^	1	1.78	6.83 × 10^−1^
							(0.43–34.40)			(0.64–60.00)			(0.11–28.63)	
		b2/b3 trip	0	1.00	–	1	–	–	0	–	–	1	–	–
		*BPY2* partial dup-Type I^*^	2	1.00	–	2	0.95	9.59 × 10^−1^	1	1.02	9.87 × 10^−1^	1	0.89	9.24 × 10^−1^
							(0.13–6.78)			(0.09–11.32)			(0.08–9.86)	
		*BPY2* partial dup-Type II^*^	2	1.00	–	1	0.47	5.43 × 10^−1^	1	1.02	9.87 × 10^−1^	0	–	–
							(0.04–5.25)			(0.09–11.32)				
		Type I^a^ + gr/gr dup^*^	0	1.00	–	1	–	–	0	–	–	1	–	–
		b2/b4 dup + gr/gr dup^*^	0	1.00	–	2	–	–	0	–	–	2	–	–
	AZFb	*RBMY1J* dup^*^	0	1.00	–	1	–	–	0	–	–	1	–	–
	AZFa	AZFa partial dup Type I^*^	4	1.00	–	1	0.24	1.97 × 10^−1^	1	0.51	5.45 × 10^−1^	0	–	–
							(0.03–2.12)			(0.06–4.57)				
		AZFa partial dup Type II^*^	0	1.00	–	1	–	–	0	–	–	1	–	–

* indicated that the CNV was not reported previously;

a *, BPY2 partial dup-Type I; dup, duplication; trip, triplication; P-values < 0.05 were considered significant*.

Two types of *BPY2* duplication (Types I and II) and “AZFa partial dup Type I” were found in both patients and controls, suggesting they might be benign CNVs. The CNVs named “*BPY2* dup Type I + gr/gr dup,” “b2/b4 dup + gr/gr dup,” “*RBMY1J* dup,” and “AZFa partial dup Type II” were all *de novo* CNVs and only identified in patient group (Table 3). Additional cases should be considered to investigate the clinical relevance of these findings.

### Distribution of Known and Novel Complex CNVs (Deletion + Duplication) in AZF Regions and Their Effects on SF

**Figure 3:**
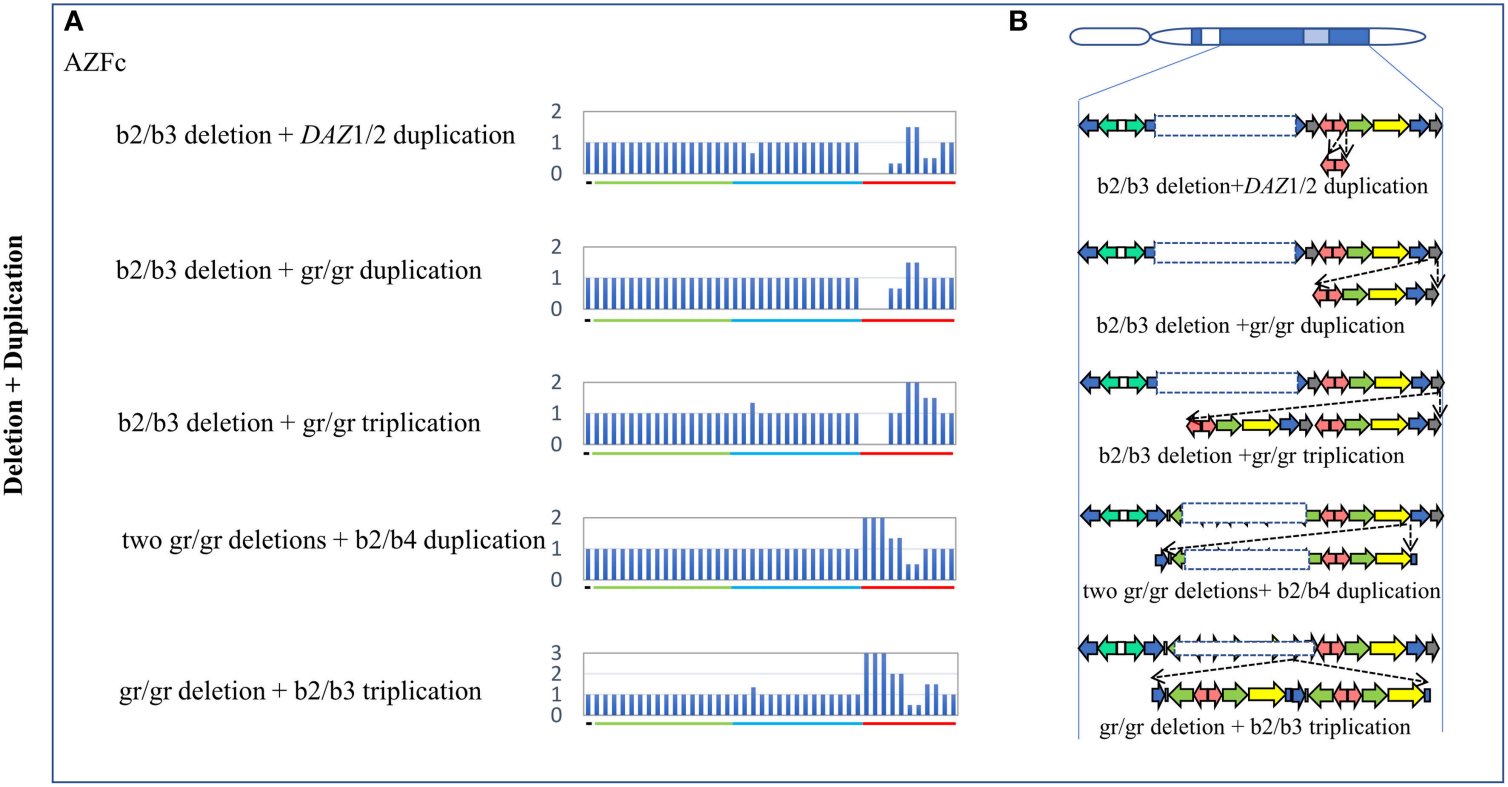
Complex CNVs (Deletion + Duplication) in AZF regions of the human Y chromosome identified by MLPA. **(A)** Results of MLPA analysis. The relative peak area of each probe was calculated by dividing the actual peak area of the subject by the average of that of five reference samples. Decreased and increased relative peak areas mean copy-number deletions and duplications, respectively. **(B)** Corresponding structures and rearrangements of AZF regions.

Five types of complex CNVs involved both deletions and duplications were identified in our study (Figure 3, Table 4). Among these complex CNVs, the “b2/b3 deletion + *DAZ* 1/2 duplication” was confirmed by analyzing the restriction enzyme digestion site of single-nucleotide variation (SNV) at sY587 to discriminate *DAZ* 1/2 from *DAZ* 3/4 (Machev et al., 2004; Supplementary Table S1). Consistent with the result reported by Lu et al. (2011), the frequency of the CNV was significantly higher in patients than that in controls (OR = 5.34, *P* = 3.00 × 10^−2^) (Table 4). The CNV named “two gr/gr deletions + b2/b4 duplications” was identified in one SF patient by Saito et al. (2015), and this CNV was considered to be benign in our patients (Table 4).

**Table 4:** Distribution of known and novel complex CNVs (Deletion+Duplication) in AZF regions and their effects on SF.

**Complex CNVs types**			**Controls**			**Patients**								
			**Normozoospermia (*****n*** **=** **402)**			**Spermatogenic failure (*****n*** **=** **423)**			**Azoospermia (*****n*** **=** **197)**			**Oligozoospermia (*****n*** **=** **226)**		
			***n***	**OR**	***P***	***n***	**OR(95%CI)**	***P***	***n***	**OR(95%CI)**	***P***	***n***	**OR(95%CI)**	***P***
Deletion + Duplication	AZFc	b2/b3 del + *DAZ*1/2 dup	2	1.00	–	11	5.34	3.00 **×10**^**−2**^	3	3.09	2.18 × 10^−1^	8	7.34	1.22 **×10**^**−2**^
							(1.18–24.24)			(0.51–18.66)			(1.55–34.87)	
		b2/b3 del + gr/gr dup^*^	6	1.00	–	8	0.81	7.10 × 10^−1^	2	0.68	6.35 × 10^−1^	6	1.80	3.14 × 10^−1^
							(0.27–2.44)			(0.14–3.39)			(0.57–5.65)	
		b2/b3 del + gr/gr trip^*^	2	1.00	–	5	2.39	2.99 × 10^−1^	0	–	–	5	4.53	7.26 × 10^−1^
							(0.46–12.04)						(0.87–23.52)	
		two gr/gr dels + b2/b4 dup	2	1.00	–	3	1.43	6.97 × 10^−1^	3	3.09	2.18 × 10^−1^	0	–	–
							(0.24–8.59)			(0.51–18.66)				
		gr/gr del + b2/b3 trip^*^	2	1.00	–	1	0.47	5.43 × 10^−1^	0	–	–	1	0.89	9.24 × 10^−1^
							(0.04–5.25)						(0.08–9.86)	

* *indicated that the CNV was not reported previously; del, deletion; dels, deletions; dup, duplication; trip, triplication; P-values < 0.05 were considered significant*.

*The bold values mean P-value < 0.05*.

In addition, three novel complex CNVs were identified in our study, including “b2/b3 deletion + gr/gr duplication,” “b2/b3 deletion + gr/gr triplication,” and “gr/gr deletion + b2/b3 triplication.” The frequency of each complex CNV showed that there was no significant difference between the patient group and the control group (Table 4).

### Comprehensive Analysis of all AZF-Linked CNVs and Their Effects on SF

Furthermore, we comprehensively analyzed all the AZF-linked CNVs to evaluate their effects on spermatogenesis. We found that “any CNV in AZF region” was at significantly higher frequency in SF cases (OR = 2.24; 95%CI, 1.62–3.08) (*P* = 8.37 × 10^−7^), azoospermia cases (OR = 1.97; 95%CI, 1.33–3.91) (*P* = 7.00 × 10^−4^), and oligozoospermia cases (OR = 2.44; 95%CI, 1.69–3.53) (*P* = 1.22 × 10^−2^), when compared with controls (Table 5).

**Table 5:** Comprehensive analysis of all AZF-linked CNVs and their effect on SF.

**CNVs types**	**Controls**			**Patients**								
	**Normozoospermia (*****n*** **=** **402)**			**Spermatogenic failure (*****n*** **=** **423)**			**Azoospermia (*****n*** **=** **197)**			**Oligozoospermia (*****n*** **=** **226)**		
	***n***	**OR**	***P***	***n***	**OR(95%CI)**	***P***	***n***	**OR(95%CI)**	***P***	***n***	**OR(95%CI)**	***P***
Any CNVs in AZF region^a^	76	1.00	–	145	2.24	**8.37** ****×**** **10**^**−7**^	62	1.97	**7.00** **×** **10**^**−4**^	82	2.44	**2.00** **×** **10**^**−6**^
					(1.62–3.08)			(1.33–3.91)			(1.69–3.53)	
Any deletions in AZF region^b^	60	1.00	–	115	2.13	**2.10** ****×**** **10**^**−5**^	49	1.89	**3.30** **×** **10**^**−3**^	65	2.30	**4.00** **×** **10**^**−5**^
					(1.50–3.01)			(1.23–2.88)			(1.55–3.43)	
Any duplications in AZF region^c^	16	1.00	–	30	1.84	5.46 × 10^−2^	13	1.70	1.65 × 10^−1^	17	1.96	6.02 × 10^−2^
					(0.99–3.43)			(0.80–3.62)			(0.97–3.96)	
Any duplications in AZFc region^d^	12	1.00	–	27	2.22	**2.47** **×** **10**^**−2**^	12	2.11	7.43 × 10^−2^	15	2.31	**3.47** **×** **10**^**−2**^
					(1.11–4.44)			(0.93–4.78)			(1.06–5.03)	

a , any CNVs (deletions or duplications or complex CNVs) in AZF region;

b , any deletions not accompanied by duplications in AZF region;

c , any duplications not accompanied by deletions in AZF region;

d *, any duplications not accompanied by deletions in AZFc region; P-values < 0.05 were considered significant*.

*The bold values mean P-value < 0.05*.

Similarly, the “any deletions in AZF region” was found at significantly higher frequency in SF cases (OR = 2.13; 95%CI, 1.50-3.01) (*P* = 2.10 × 10^−5^), azoospermia cases (OR = 1.89; 95%CI, 1.23–2.88) (*P* = 3.30 × 10^−3^), and oligozoospermia cases (OR = 2.30; 95%CI, 1.55–3.43) (*P* = 4.00 × 10^−5^), when compared with controls (Table 5).

For “any duplications in AZFc region”, the frequency was significantly higher in SF cases (OR = 2.22; 95%CI = 1.11–4.44) (*P* = 2.47 × 10^−2^) and oligozoospermia cases (OR = 2.31; 95%CI = 1.06–5.03) (*P* = 3.47 × 10^−2^) than controls (Table 5).

### Comparing MLPA Method With Conventional STS-PCR

**Figure 4:**
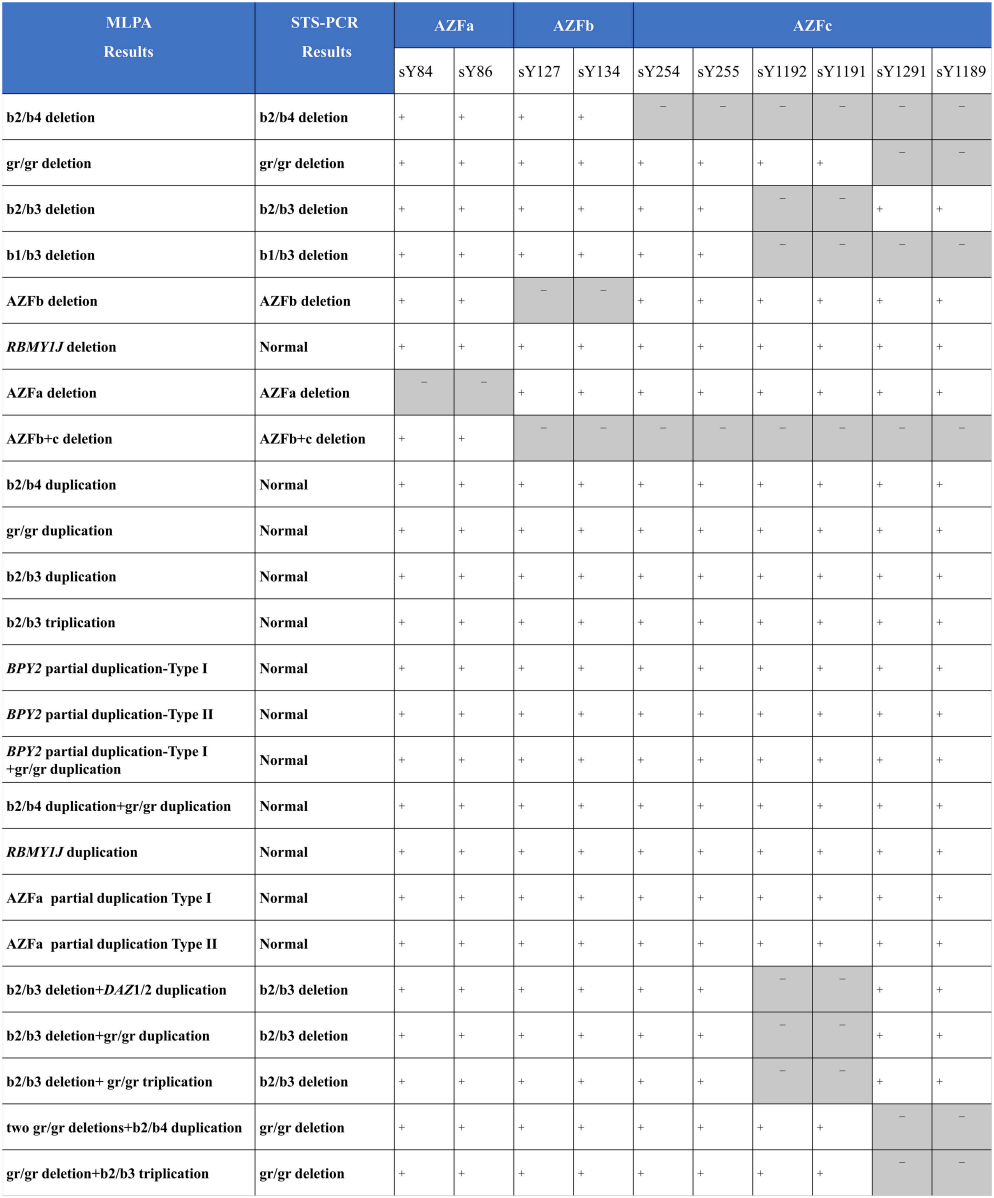
Comparing MLPA methods with traditional agarose electrophoresis for STS markers. +, the fragment in the STS site was not deleted; -, the fragment in the STS site was deleted.

As compared with MLPA, only seven known deletions (29.2%, 7/24) were identified by STS-PCR. One deletion (4.2%, 1/24) and eleven duplications (45.8%, 11/24) were not identified, and five complex CNVs (20.8%, 5/24) were detected as simple deletions (Figure 4), demonstrating that the MLPA method possesses more powerful efficiency in detecting AZF-linked CNVs.

### The qPCR Validation of CNVs Not Confirmed by STS-PCR

To validate the CNVs not verified by STS-PCR especially those AZF-linked duplications and complex CNVs, we performed real-time quantitative PCR (qPCR).

**Figure 5:**
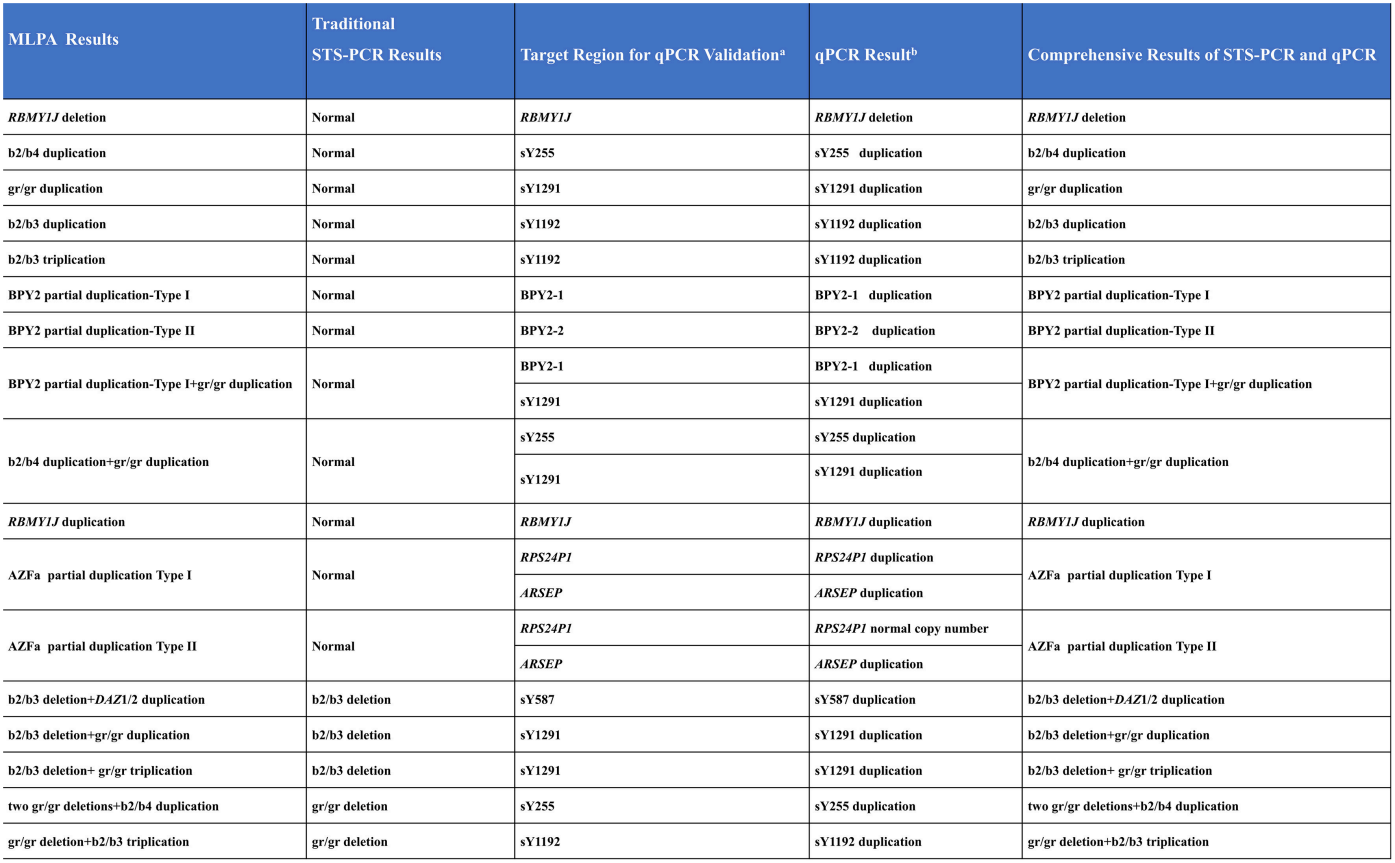
Comprehensive results of STS-PCR and qPCR validation. ^a^, Special regions of target AZF-linked CNVs for qPCR validation; ^b^, Simplified qPCR results. Detailed qPCR results see Supplementary Figure S2.

For each CNV to be tested, two reference samples (normal MLPA results) and one randomly selected positive CNV sample were examined. The qPCR results were all consistent with MLPA results (Figure 5 and Supplementary Figure S2). Those above results indicated the accuracy of the MLPA method for detecting AZF-linked CNVs ( both deletions and duplications).

## Discussion

Y chromosome microdeletion was the second most frequent genetic etiology after Klinefelter syndrome (Krausz et al., 2014). In recent years, AZFa, b and c (b2/b4) deletions have been identified as the pathogenic CNVs in spermatogenesis (Krausz et al., 2014). However, AZF-linked duplications and complex CNVs were rarely studied due to limits of technical means. In this study, we revealed the relatively precise frequency and clinical significance of AZF-linked CNVs in Han Chinese population by using the MLPA method.

For AZFc partial deletions (gr/gr del, b2/b3 del, and b1/b3 del), we did not observe significantly different distributions between SF cases and control subjects. The clinical significance of these partial deletions was reported to be variable in previous studies (Lu et al., 2009; Bansal et al., 2016; Krausz and Casamonti, 2017). Hence, further study should be performed in diverse racial or ethnic groups to confirm the respective results.

Interestingly, two *de novo RBMY1J* CNVs, a deletion and a duplication were identified in our oligozoospermic patients. *RBMY1J*, a member of *RBMY1* (Y-linked RNA binding motif protein family 1), is expressed in all stages of spermatogenesis and acts as a splicing factor during spermatogenesis (Elliott et al., 1997; Dreumont et al., 2010). Yan et al. revealed that *RBMY1* is associated with sperm motility (Yan et al., 2017). Considering the normal sperm motility in both patients, our study suggests that *RBMY1J* is associated with decreased sperm numbers in clinic. Therefore, further studies are required to determine the function of *RBMY1* in spermatogenesis.

Besides, Y chromosome is thought to be “fragile” owing to complex NAHR structure. In the present study, “any CNVs in AZF region” showed significantly higher frequency in SF patients than controls, and we speculated that the instability of Y chromosome might be associated with spermatogenic impairment. Additionally, the frequency of “any duplication in AZFc region” was also significantly higher in SF patients, suggesting that AZFc-linked duplications might play vital roles in spermatogenesis. Similar results were reported in the previous studies (Lin et al., 2007; Ye et al., 2013; Saito et al., 2015).

In addition, three novel complex CNVs identified in the present study expanded our knowledge on AZF-lined CNVs. Notably, all five complex CNVs were detected as simple deletions (gr/gr or b2/b3 deletion) by the traditional STS-PCR method. The technical limitation of STS-PCR would be a potential confounding factor for risk assessment in studies of gr/gr or b2/b3 deletion. It justifies why the previous research has demonstrated conflicting findings.

In 2012, Bunyan et al. performed MLPA in 100 subjects (50 SF patients and 50 controls) and identified four types of simple deletions, which cannot be detected by STS-PCR (Bunyan et al., 2012). In 2014, Liu et al. performed MLPA in 199 fathers and their 228 sons, and found that assisted reproductive technology didn't increase the risk of Y-chromosome microdeletions in male offspring (Liu et al., 2014). Both studies used the MLPA analysis to detect Y-chromosome microdeletions. In 2015, Saito et al. performed MLPA in 56 SF patients and 65 control individuals from who all were Japanese, and identified both AZF-linked deletions and duplications (Saito et al., 2015). Compared with previous studies, we applied the MLPA analysis to more subjects and identified more types of AZF-linked CNVs. Besides, for those novel CNVs only detected in SF patients, we further detected if they were inherited from their fathers and found they were all *de novo* CNVs. In addition, we classified the patients with SF into azoospermia group and oligozoospermia group, which would be helpful to understand the clinical significance of AZF-linked CNVs in different SF types.

However, there are some limitations in the current study. Although a decent number of subjects were tested in this retrospective case-control study, it is not enough to reach conclusions on the role of those rare AZF-linked CNVs. In addition, the *p*-values presented were not corrected for multiple testing in the statistical analysis, which may lead to reject the null hypothesis too readily and increase the risk of false positives. In the future, we plan to establish the roles of rare AZF-linked CNVs in a larger prospective study.

In summary, this study comprehensively identified AZF-linked CNVs, especially duplications, in Han ethnicity. It provides valuable findings for understanding the roles of AZF-linked CNVs in spermatogenesis, and will be helpful in the diagnosis and treatment of SF in clinical practice.

## Ethics Statement

This study was carried out in accordance with the recommendations of Nanjing Maternity and Child Health Care Hospital with written informed consent from all subjects. All subjects gave written informed consent in accordance with the Declaration of Helsinki. The protocol was approved by the Ethics Committee of Nanjing Maternity and Child Health Care Hospital.

## Author Contributions

RZ and JC performed the experiments and wrote the manuscript. DM and JT analyzed the data. YW and PH performed clinical evaluation and collected clinical data. ZX designed the study.

### Conflict of Interest Statement

The authors declare that the research was conducted in the absence of any commercial or financial relationships that could be construed as a potential conflict of interest.
