# Supplementary material for: Identifying Novel Copy Number Variants in Azoospermia Factor Regions and Evaluating Their Effects on Spermatogenic Impairment
Source: Front Genet. 2019 May 7;10:427. doi: 10.3389/fgene.2019.00427 (PMC6514098; doi:10.3389/fgene.2019.00427)
Supplement: Supplementary file 1 [file Data_Sheet_1.PDF]

Supplementary Material

Identifying novel copy number variants in azoospermia factor regions and evaluating their effects on spermatogenic impairment

Ran Zhou†, Jian Cheng†, Dingyuan Ma, Jianxin Tan, Yuguo Wang, Ping Hu and Zhengfeng Xu*

*** Correspondence:** Zhengfeng Xu: zhengfeng_xu_nj@163.com

1 Supplementary Figures and Tables

1.1 Supplementary Figure

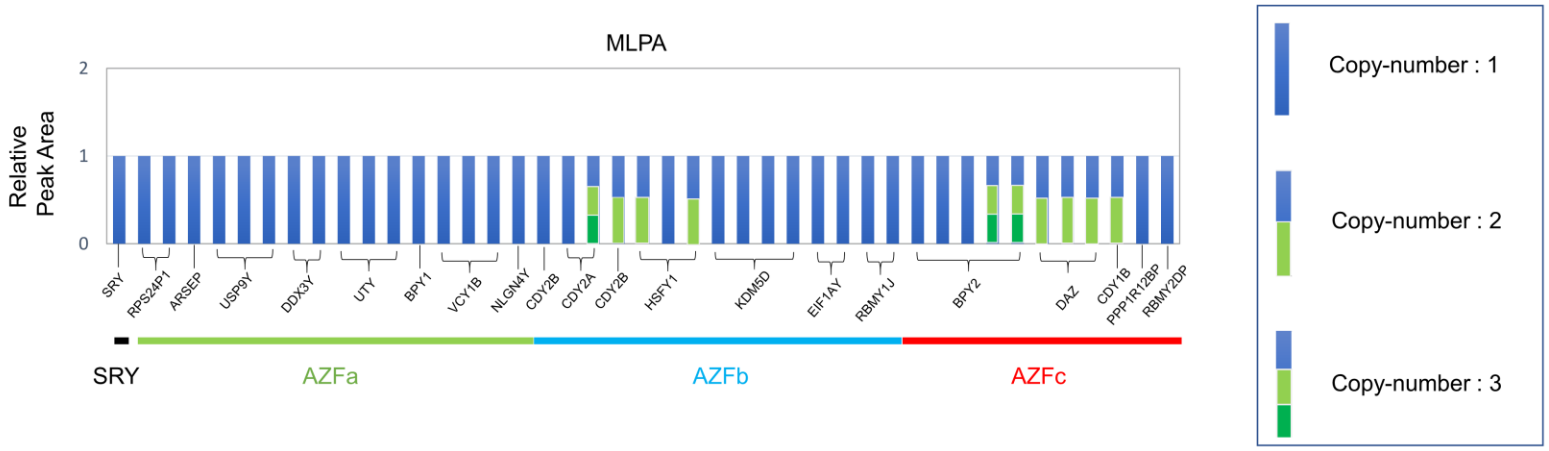

Supplementary Figure S1. Probes distribution in MLPA in the present study. This kit contained 42 specific probes for AZF regions.

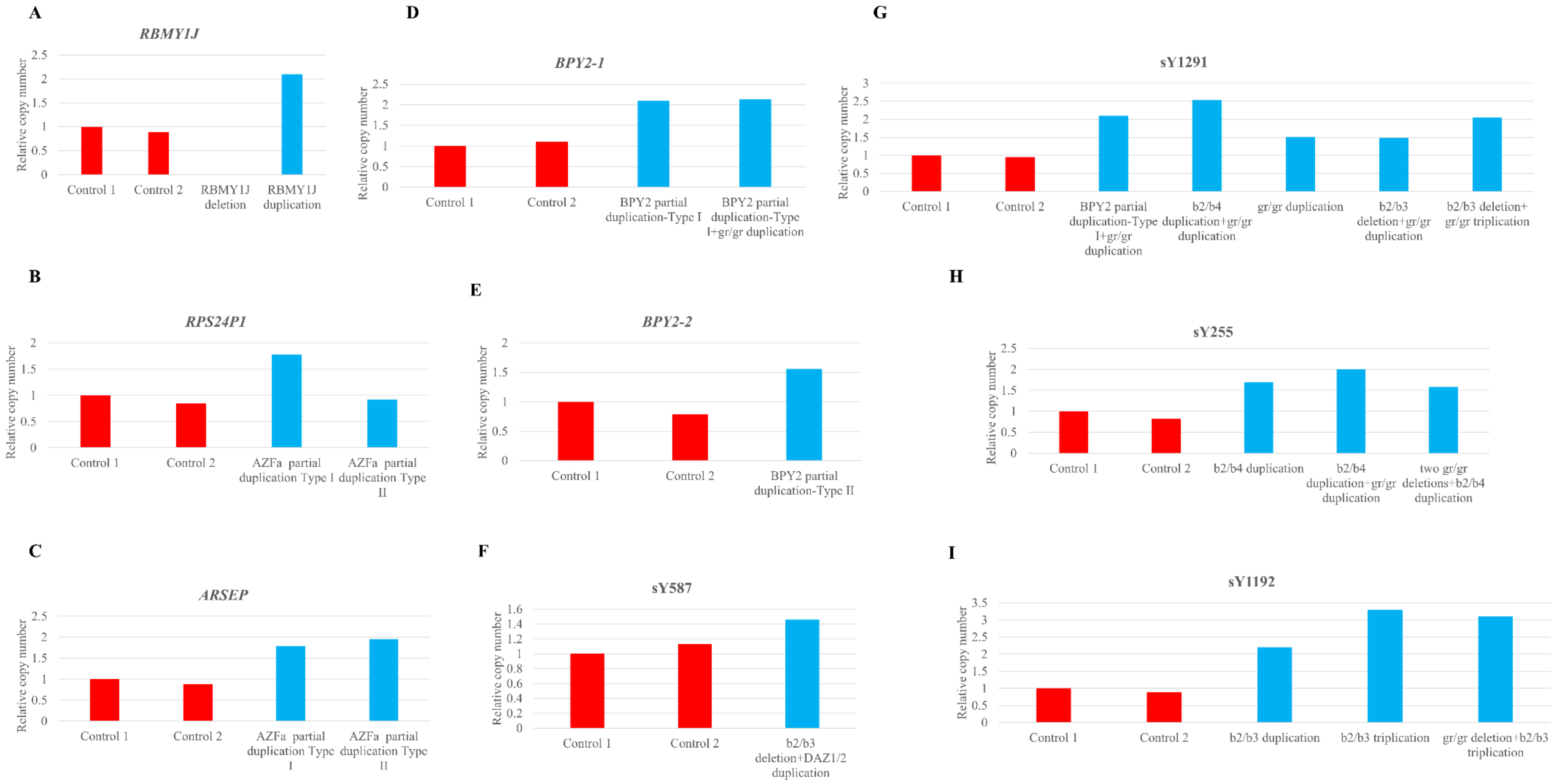

Supplementary Figure S2. The qPCR validation results of CNVs not comfirmed by STS-PCR. For each CNV to be tested, two reference samples (normal MLPA results) and one randomly selected positive CNV sample were examined. The copy number of other samples were

normalized against the normal Control 1. qPCR results of *RBMY1J* deletion and *RBMY1J* duplication (A), AZFa partial duplication Type I and II (B-C), *BPY2* partial duplication-Type I and II, *BPY2* partial duplication-Type I+gr/gr duplication (D-E), b2/b3 deletion+*DAZ*1/2 duplication (F), *BPY2* partial duplication-Type I+gr/gr duplication, b2/b4 duplication+gr/gr duplication, gr/gr duplication, b2/b3 deletion+gr/gr duplication, b2/b3 deletion+ gr/gr triplication (G), b2/b4 duplication, b2/b4 duplication+gr/gr duplication, two gr/gr deletions+b2/b4 duplication (H), b2/b3 duplication, b2/b3 triplication, gr/gr deletion+b2/b3 triplication (I).

1.2 Supplementary Table

Supplementary Table S1. *DAZ* gene copy deletion pattern and STS information for b2/b3 deletion only and b2/b3 del+*DAZ*1/2 dup.
a, b2/b3 deletion not accompanied by duplication; del, deletion; dup, duplication.

**CNVs Type**	**STS**						**Deleted *DAZ* copies**	***DAZ* gene copies**
	**sY254**	**sY255**	**sY1192**	**sY1191**	**sY1291**	**sY1189**	**(sY587)**	
b2/b3 deletion only[a]	+	+	-	-	+	+	*DAZ*1/2 or *DAZ*3/4	2
b2/b3 del + *DAZ*1/2 dup	+	+	-	-	+	+	*DAZ*3/4	>4

Supplementary Table S2. Primer sqeuence for qPCR.

Primer	Forward Sequence (5'-3')	Reverse Sequence (5'-3')
ACTB	GCCATGTACGTTGCTATCCA	CCTCGTAGATGGGCACAGT
RBMY1J	GGGCCTCGGATGTCTTATGG	CTTCACGAGGATCAGGGAGC
sY255	GTTACAGGATTCGGCGTGAT	CTCGTCATGTGCAGCCAC
sY1291	TAAAAGGCAGAACTGCCAGG	GGGAGAAAAGTTCTGCAACG
sY1192	ACTACCATTTCTGGAAGCCG	CTCCCTTGGTTCATGCCATT
BPY2-1	ATCCGGCTTCCAGAAATGGT	AACTGAAACTCCCCAGTGGC
BPY2-2	TTCAGGGTCTTTGTATTCATGCC	GTTTGTGCTACCAAAAAGCAGC
RPS24P1	ATCCCAGGGAAACAGCAAGT	CCAGAACCAACAGTGGCCTT
ARSEP	GACCCACATCCTCACACCAG	GCACTAGCAGAGGGGGAATG
sY587	TGGTTAATAAAGGGAAGGTGTTTT	TCTCCAGGACAGGAAAATCC
